# Study Approach of Antioxidant Properties in Foods: Update and Considerations

**DOI:** 10.3390/foods6030017

**Published:** 2017-02-28

**Authors:** Alessandra Durazzo

**Affiliations:** Consiglio per la ricerca in agricoltura e l’analisi dell’economia agraria, Centro di Ricerca CREA-Alimenti e Nutrizione, Via Ardeatina 546, 00178 Roma, Italy; alessandra.durazzo@crea.gov.it; Tel.: +39-065-149-4499

**Keywords:** antioxidant properties, extractable compounds, non-extractable compounds, pure compounds, biologically active fractions, study approach

## Abstract

The assessment of interactions between natural antioxidants and other food matrix components represents the main step in the investigation of total antioxidant properties, in terms of potential health benefits. The diversity of chemical structures of natural compounds, besides their possible interactions, as well as the biological role and different modes of action makes it difficult to assess a single and reliable procedure for the evaluation of antioxidant activity. Today, much attention is given to the distinction between extractable and non-extractable antioxidants as a key tool in the description of the nutritional and healthy properties of food matrices. The starting point for the investigation of antioxidant effects of food extracts is the analysis of antioxidant properties of pure compounds and their interactions. Another complementary approach could be represented by the study of how different biologically active compound-rich extracts contribute to the total antioxidant capacity.

## 1. Main Phases of Study of Antioxidant Properties: Focus on Extraction Procedure

The antioxidant properties of each food matrix come from the combined and concerted action of biologically active compounds, i.e., polyphenols, carotenoids, lignans, glucosinolates, etc. Antioxidants can exert large spectra of biological and physiological functions, i.e., anti-allergic, anti-atherogenic, anti-inflammatory, antimicrobial, antioxidant, anti-thrombotic, cardioprotective, etc. [1]. The identification and quantification of the content of antioxidants as well as the understanding and assessment of interactions between these biologically active compounds and other food matrix components could be seen as the main step in the investigation of total antioxidant properties, in terms of both the potential health benefits of food and as the indicator of a “possible beneficial role”.

The diversity of chemical structures of natural compounds, besides their possible interactions, as well as the biological role and the different modes of action makes it difficult to assess a single and reliable procedure for the evaluation of antioxidant activity.

Three essential items should be identified and developed in the evaluation of antioxidant properties: the extraction procedure, the antioxidant capacity measurements and the expression of results [2,3,4,5,6].

Chemical extraction represents an important issue. It is influenced by the types of solvents, extraction time and temperature, as well as by the chemical composition and physical characteristics of the analyzed sample [7]. There are some main aspects to consider in order to improve the recovery of antioxidants. For this reason, some authors have tested the use of different times and temperatures [8,9,10] or different solvents, i.e., ethanol, methanol, acetone and/or mixtures thereof [11,12], that have different extraction efficiencies. For this purpose, Celik et al. [13] discuss the importance of the solvents’ effects on the extraction recovery as well as on the performance of antioxidant assays.

Normally, it appears that the use of an acid mixture of methanol/water improves the extraction efficiency since it allows us to obtain extracts with the highest level of antioxidants [14,15]. Moreover, recent investigations have also performed alkaline hydrolysis, acid hydrolysis or enzymatic digestion [16,17,18,19,20,21,22].

In recent years, much attention has been given to the distinction between extractable and non-extractable antioxidants [23,24,25] as a key tool in the description of nutritional and healthy properties of food matrices.

## 2. Extractable Antioxidants and Non-Extractable Antioxidants: Some Examples

Antioxidants occur in two forms: on the one side, as easily extractable compounds (free forms), made soluble by aqueous-organic solvents; on the other side, as less extractable compounds that are in bound forms because they remain in the residue after aqueous-organic extract. In detail, non-extractable antioxidants are linked to the food matrix by covalent bonds, hydrogen bonds and/or hydrophobic interactions. Non-extractable antioxidants encompass hydrolysable tannins and other classes, i.e., phenolic acids and hydroxycinnamic acids, bound to carbohydrates and proteins as well as macromolecules, such as condensed tannins (proanthocyanidins) [23].

The antioxidant properties of plant foods have been underestimated: significant amounts of bioactive compounds remain in the residue from extraction as non-extractable antioxidants. Their incidence in foodstuffs and diet were studied because they represent a significant portion in some groups of foods, i.e., cereals, fruits, vegetables, nuts, and legumes [25,26,27,28]. Some specific and different hydrolytic treatments of the residue were developed and performed for the isolation of non-extractable compounds related to the food item within each food group, the type of bonds with the food matrix as well as the nature and structure of the target compounds [23,25,28]. In relation to the presence of multiple aspects and factors, it becomes difficult to carry out a categorization of the main trends of the contribution of extractable and non-extractable antioxidants to the total antioxidant properties of the major food groups. For this reason, some relevant examples were reported. Generally, however, researchers concluded and remarked that the analysis of antioxidants in plant foods that remain in the residues was necessary and required for a comprehensive and appropriate identification of antioxidant properties. As for this aspect, studies are still needed to tackle this issue. Moreover, these studies should also be combined with an in vitro physiological approach, and together they will represent the basis for in vivo studies of antioxidant mechanisms [29,30].

Among the examples of studies concerning fruit items [26,31,32], Kristl et al. [32] studied the evaluation of the contribution of extractable and non-extractable compounds to the total antioxidant activity of plums. In this paper, in addition to the study of extractable compounds, hydrolyzable tannins and non-extractable proanthocyanidins from residues by two different acidic treatments were investigated: extractable antioxidants contributed less than 18% to the total antioxidant activity, whereas non-extractable compounds contributed more, according to what was previously discussed by other authors in relation to hydrolyzable tannins and/or non-extractable proanthocyanidins [26,33] for fruit items. In detail, Arranz et al. (2009), by studying extractable polyphenols, hydrolyzable polyphenols and proanthocyanidins in apple, peach, and nectarine, showed that the non-extractable polyphenols content (112–126 mg/100 g of fresh fruit) was higher than the extractable polyphenols content (18.8–28 mg/100 g of fresh fruit).

Considerable research was carried out both on grains [34,35,36] and their derivatives, i.e., bread, pasta [37,38] as well as on pulses [25]. In the context of such research, the work by Durazzo et al. [37] was focused on the antioxidant properties of experimental pastas made with different wholegrain cereals in raw and cooked products; here, the authors found that in cooked pasta, FRAP (Ferric Reducing Antioxidant Power) values ranged from 3.26 ± 0.08 μmol/g dry weight to 19.52 ± 1.28 μmol/g dry weight in aqueous-organic extracts and from 17.91 ± 2.83 μmol/g dry weight to 87.83 ± 5.06 μmol/g dry weight in residues [37].

With regards to fatty food matrices, it is interesting to mention the work of Arranz et al. [39] who studied the contributions of two major fractions of walnut (oil and defatted matter) on antioxidant properties and then compared it to the data obtained from the analysis of whole walnut for better understanding the possible interference of oil in the antioxidant properties: the contribution of walnut oil to the total antioxidant capacity of walnut was less than 5% and hydrolyzable tannins represented the main contributors to the antioxidant properties in the defatted matter. In addition to this, the authors recommended a separate determination of antioxidant capacity in the oil and defatted matter of walnut [39]. Rufino et al. [40] studied the antioxidant activity of a tropical fruit with a fat content of about 20% by taking into consideration its defatted matter and oil and paying particular attention to the polar and apolar fractions obtained from oil extraction. Generally, further research is needed in this direction.

Considering that only small amounts of vegetables are consumed raw, whereas most food is consumed after being cooked or processed, it is important to study the effect of processing on antioxidant properties [41,42] as well as the antioxidant properties of complex food matrices and ready-to-eat dishes that take into account both the formulation and the technological process [43,44]. In particular, Durazzo et al. [45], by studying the antioxidant properties of some traditional Italian dishes, have shown that in spaghetti alle vongole, pomodori al riso, gateau di patate and pan di Spagna, extractable polyphenols contributed less than 15% to the total antioxidant activity, while hydrolyzable polyphenols gave a major contribution. Carciofi alla romana, instead, showed an inverse trend.

It is worth mentioning the recent investigations on extractable and non-extractable antioxidants of fruit waste [46,47,48,49] and vegetable waste [50] as examples of innovative applications of studies on antioxidants in circular bioeconomy and biorefinery.

## 3. Two Complementary Approaches: From the Monitoring of the Antioxidant Behavior of Standard Compounds and Their Interactions to the Study of Contributions of Biologically Different Active Compound-Rich Extracts to Total Antioxidant Capacity

The investigation of antioxidant effects of food extracts starts with the analysis of the antioxidant properties of pure compounds, i.e., terpenoids, carotenoids, limonoids, phytosterols, glucosinolates, polyphenols, flavonoids, isoflavonoids and anthocyanidins and/or their mixtures. Targeted investigations were carried out on the antioxidant properties of standards or reference compounds for the evaluation of food extracts, with particular attention also paid to the comparison of different classes of compounds [51,52,53,54,55,56]. In regards to this, the study of Tabart et al. [56] that compares the antioxidant properties of standard compounds, i.e., phenolic compounds, ascorbic acid, and glutathione as measured by various assays is interesting.

In the context of such investigations, we reported, as examples, the case study of lignans and glucosinolates, two classes of compounds that have a different range of antioxidant properties.

Lignans and their active metabolites are known for their structure which is similar to that of estrogenic compounds; several authors have reported differences in antioxidant properties between plant lignans and enterolignans [25,55,57]. Niemeyer and Metzler, [55] hypothesized that the observed differences are presumably related to the methoxy group. Eklund et al. [57], by monitoring the radical-scavenging activity using DPPH (2,2-diphenyl-1-picrylhydrazyl), showed and concluded that compounds with catechol (3,4-dihydroxyphenyl) moieties had the highest radical-scavenging activity.

The research of Durazzo et al. [25], by monitoring antioxidant properties by means of FRAP assay, found not only significantly higher FRAP values in plant lignans compared to enterolignans, but also noticed the highest concentration of antioxidant properties in matairesinol.

With regards to glucosinolates, Barillari et al. [58] have studied the direct antioxidant activity of purified glucoerucin, isolated and purified from rocket seeds and sprouts: glucoerucin and its metabolite erucin possess good direct antioxidant activity as hydroperoxide-scavenging preventive antioxidants. Flavonoid and hydroxycinnamic acids are responsible for antioxidant properties in cruciferous vegetables rather than their main class of compounds, glucosinolates [59]. The recent investigation of Natella et al. [60], which, by studying the in vitro redox behavior of 15 glucosinolates by means of different methodologies, is interesting; it showed that sinalbin and gluconasturtiin were highly active in scavenging ABTS radicals and in protecting LDL (low-density lipoprotein) from copper-catalyzed oxidation and concluded that few glucosinolates can act as antioxidants. Further research should be conducted on investigating the antioxidant capacity of glucosinolates and their metabolites.

The core element is the evaluation of concerted and synergistic actions, of antagonist interactions or of no effect among active compounds that depends both on the nature and the peculiar combination of antioxidants and on the complex structure of the food matrix [25,61,62,63].

Reber et al. [62] investigated the antioxidant capacity of the interactions and the chemical/structural model of phenolic compounds found in strawberries: the antioxidant capacity of a whole fruit exceeds the sum of the single antioxidant values in the fruit, and this underlies the presence of the synergistic effect. Moreover, the study of the interactions among seven phenolic compounds (p-coumaric acid, cyanidin, catechin, quercetin-3-glucoside, kaempferol, pelargonidin and ellagic acid), taking into account the relative concentrations found in strawberries, underlined synergism or antagonism in relation to the combination studied.

Palafox-Carlose et al. [63] have studied the interactions of four major phenolic compounds (chlorogenic, gallic, protocatechuic and vanillic acid) found in “Ataulfo”: there was a synergistic interaction between the studied phenolic compounds, except for vanillic acid, which appears to have a negative effect.

Besides the evaluation of the contribution of extractable and non-extractable to total antioxidant properties, an interesting and alternative study approach is represented by the isolation of biologically active compound-rich extracts of each food matrix (i.e., limonoid-rich extracts, anthocyanin-rich extracts, etc.) and the evaluation of their contributions to the total antioxidant capacity; one or more fractions for each food can be identified and minor or major contributors to the antioxidant properties can be highlighted.

The study of Mattera et al. [64] evaluated the radical-scavenging activity of a fat-soluble vitamins–rich extract in an Italian cheese, related to the structure of the active substances present in lipophilic extracts and their possible interactions, as an indicator of the antioxidant properties of cheese.

Durazzo et al. [65], by investigating the bioactive components of commercial carob flours, have reported that the antioxidant properties found by means of the FRAP assay in lignan-rich extracts matched those obtained from aqueous-organic extract and residue.

## 4. Conclusions

Studies on the evaluation of antioxidant properties should be integrated into a multidisciplinary system of analysis, coupled with chemometrics, in order to develop an innovative study approach for food research. This system should also include “green” procedures as Fourier transformed infrared spectroscopy (FTIR) on attenuated total reflectance (ATR), etc. The studies on the identification of extractable and non-extractable antioxidants represent the basis for further studies on the biological role of these specific isolated fractions [48,66], in order to increase the application and uses of nutraceutical and functional foods.
